# Interference Confocal Microscope Integrated with Spatial Phase Shifter

**DOI:** 10.3390/s16091358

**Published:** 2016-08-24

**Authors:** Weibo Wang, Kang Gu, Xiaoyu You, Jiubin Tan, Jian Liu

**Affiliations:** 1 Institute of Ultra-precision Optoelectronic Instrument Engineering, Harbin Institute of Technology, Harbin 150001, China; weibo.wang@eng.ox.ac.uk (W.W.); gukang90@163.com (K.G.); youxiaoyudyx@163.com (X.Y.); jbtan@hit.edu.cn (J.T.); 2 Department of Engineering Science, University of Oxford, Parks Road, Oxford OX1 3PJ, UK

**Keywords:** confocal microscope, interference, phase shift, spatial phase shifter

## Abstract

We present an interference confocal microscope (ICM) with a new single-body four-step simultaneous phase-shifter device designed to obtain high immunity to vibration. The proposed ICM combines the respective advantages of simultaneous phase shifting interferometry and bipolar differential confocal microscopy to obtain high axis resolution, large dynamic range, and reduce the sensitivity to vibration and reflectance disturbance seamlessly. A compact single body spatial phase shifter is added to capture four phase-shifted interference signals simultaneously without time delay and construct a stable and space-saving simplified interference confocal microscope system. The test result can be obtained by combining the interference phase response and the bipolar property of differential confocal microscopy without phase unwrapping. Experiments prove that the proposed microscope is capable of providing stable measurements with 1 nm of axial depth resolution for either low- or high-numerical aperture objective lenses.

## 1. Introduction

For the non-contact 3D measurement of Micro-Electro-Mechanical System (MEMS) and micro-optics, both deep micro-topographic structures and surface roughness have to be controlled during the same measurement procedure, preferably with the same instrument. The need for a highly-resolved metrological 3D measuring system having a large height dynamic range and high axis resolution becomes crucial. Confocal Microscopy has a very large dynamic range and an optical sectioning capability of less than 1 to 2 microns [1,2], and Phase Shift Interferometry (PSI) has a very high resolution inside a very short dynamic range. Interference confocal microscopy (ICM) can be built while combining the respective advantages of the interferometry and confocal microscopy [3,4] to obtain high axis resolution and large dynamic range similar to coherence scanning interferometry (CSI)—e.g., Bruker’s Contour Elite 3D Optical Microscope (combining vertical resolution with a sub-nanometer to greater than 10 mm vertical range) and ZYGO’s NewView™ 7000 Series surface profiling system (Surface Topography Repeatability (STR) is sub-nanometer, Max. Step Height is up to 20 mm), but higher lateral resolution due to the property of confocal microscopy. Moreover, the basic property of optical sectioning inherent to confocal imaging is particularly well-adapted to PSI, since it automatically solves the critical and time consuming problem of phase unwrapping computation [4].

Commonly, two optical sectioning methods are used in confocal microscopy. One simply detects the peak position of the axial intensity response, e.g., differential confocal microscopy [5]. The other method, bipolar differential confocal microscopy—with its bipolar property and absolute zero tracking feature—can be used to improve the peak detection sensitivity (which is low in the former) and expand the linear range to two times as large as that in conventional confocal microscopy [6]. Conventional confocal microscopy is sensitive to the reflectivity changing of the measured surface and the vibration of the environment. Some methods have been employed to remove the ambiguity caused by the changes in reflectivity [7,8]. For example, in the Olympus LEXT OLS4100 microscope, a dual-detector scheme is used to remove the effects of reflectivity changes on the surface [9].

Interference confocal microscopy is another effective technique to improve the axial depth resolution and remove the ambiguity caused by the changes in reflectivity [3,10]. Confocal microscopy can be merged with phase-shifting interferometry based on optical path modulation [10,11] and wavelength modulation [12], which are always time-consuming and structure complicated in the time-dependent phase shift method and the spatial phase shift method, respectively.

We present a phase-shifting interference confocal microscope using a novel single-body four-step phase-shifter for phase demodulation. It is presented by introducing a compact single body spatial phase-shifter and interference structure in bipolar differential confocal microscopy. The proposed ICM combines with bipolar differential confocal microscopy to obtain large range and long working distance and a simultaneous phase shifting module to reduce the sensitivity to vibration and reflectance disturbance seamlessly. Experiments are implemented for verification of the axial depth resolution.

## 2. Methods

**Figure 1:**
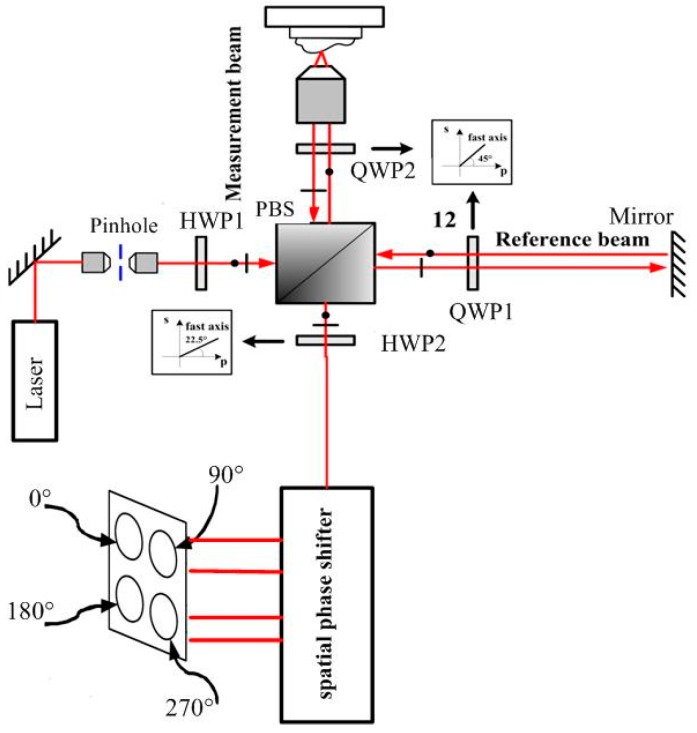
Schematic diagram of a phase-shifting interference confocal microscope using the herein reported new single-body four-step phase-shifter phase-demodulator. HWP1: half wave phase plate; PBS: polarized beam splitter; QWP: quarter wave phase plate.

The interference confocal microscope (ICM) merges a typical optical layout of bipolar differential confocal microscopy with a compact single body spatial phase shifter and interference structure for simultaneous phase shift, as shown in Figure 1. The compact single body spatial phase-shifter is introduced to replace the previous complex phase-shifter devices and get a stable and space-saving, simplified system. The proposed ICM can obtain reduced sources of error, and be easy to align.

**Figure 2:**
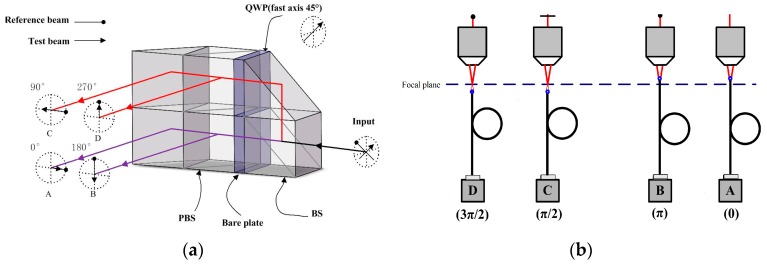
New single-body four-step phase-shifter for simultaneous phase-demodulation. (**a**) single body spatial phase shifter; (**b**) detectors with differential confocal microscopy.

The incident light beam is linearly polarized in a plane at 45°. Because of the different reflectivity of the test surface and the reference surface, a half wave phase plate (HWP1) was inserted before the polarized beam splitter (PBS) to maximize the interference signal contrast. The interferometer module is like the Twyman–Green interferometer, with a polarizing cube beam splitter. When the transmitted and the reflected light go to the reference surface and the test surface, both beams pass twice through quarter wave phase plates (QWP) with their axes at 45° before returning to the beam splitter. Thus, both planes of polarization will rotate by 90°. This allows the returning beams to go to the observing screen instead of returning to the light beam [13,14]. The reference beam and test beam are combined by the PBS, and forwarded to HWP2 and the single body spatial phase shifter. The details of the spatial phase-shifter are redrawn in Figure 2. The optical design primarily follows the concept of [15,16]. All the essential optical components are integrated together into a single body to increase the robustness against vibration. Through the spatial phase-shifter, both the reference beam and the test beam are divided to four beams with phase shifts of 90° between each other simultaneously without time delay. So, the effect of vibration can be avoided observably.

The polarized direction of the reference beam is rotated to be 45° with respect to the horizontal axis but perpendicular to the test beam before entering the spatial phase shifter by the λ/2 phase plate. Then, two beams are divided into two paths by the PBS. The PBS in the lower path produces two interference patterns with the phase shifts 0° and 180° between the reference and test beams. In the upper path, only the test beam is phase shifted by 90° using a QWP before interference. So, the two interference patterns resulting from the upper polarization beam splitter consequently yield phase shifts of 90° and 270°, respectively [16]. The four interference signals have phase shifts of 90° between each other simultaneously without time delay, and are concurrently captured with four detectors. Then, the four interference signals are processed by the four-bucket algorithm.

The detectors are placed in bipolar differential confocal microscopy modes, as shown in Figure 2b. Two interference signals are collected by objectives (NA = 0.1) and single mode fibers (*d* = 5 μm), whose input ends are located at the defocused displacement −*u_d_* before the focal plane as pinholes, and are then detected by photodetectors (A,B). The other two interference signals are collected by the same types of objectives and single mode fibers, whose input ends are located at the defocused displacement +*u_d_* after the focal plane, and then detected by photodetectors (C,D), respectively. Finally, four channels of interference signal with a 90° phase shift between each other are thus produced.

The differential intensity signal *I_diff_* can be derived from

(1) $$I_{diff}(u)=[I_{C}(u)+I_{D}(u)]-[I_{A}(u)+I_{B}(u)]=2S^{2}\left[{\mathrm{sinc}}^{2}\left(\frac{2u+u_{d}}{4\pi }\right)-{\mathrm{sinc}}^{2}\left(\frac{2u-u_{d}}{4\pi }\right)\right]$$

where *u* is the dimensionless axial coordinate.

Differential phase signal *I_phase_* is calculated by

(2) $$I_{phase}(u)=\frac{I_{C}(u)-I_{D}(u)}{I_{A}(u)-I_{B}(u)}=\frac{I_{a}^{-u_{d}}(u)}{I_{a}^{+u_{d}}(u)}\tan[\Delta \varphi (u)]=K_{u_{d}}(u)\cdot \tan[\Delta \varphi (u)]$$

where $K_{u_{d}}(u)$ is called the correction factor, expressed as

(3) $$K_{u_{d}}(u)=\mathrm{sinc}\left(\frac{2u-u_{d}}{4\pi }\right)/\mathrm{sinc}\left(\frac{2u+u_{d}}{4\pi }\right)$$

It can be seen from Equations (2) and (3) that there are no light intensity signals existing in the interference confocal microscope, implying that the test result is not sensitive to the disturbance of the intensity of a reflected beam and the vibration of the environment.

**Figure 3:**
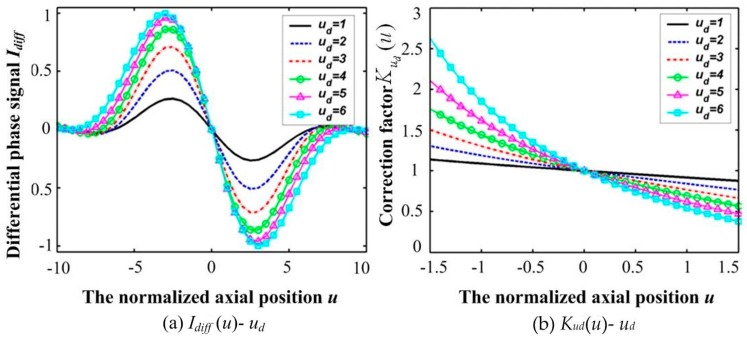
Variations in *I_diff_*(*u*) and *K*(*u_d_*) responding to various defocused quantities *u_d_*.

Variations in *I_diff_*(*u*) and *K*(*u_d_*) responding to various defocused quantities *u_d_* are shown in Figure 3, which obviously shows the bipolar property of differential confocal microscopy. Clearly, the sign of *I_diff_* changes only in the main cycle of *I_phase_*. It is important to determine the main cycle of *I_phase_*, within which it is monotonically increasing with *u*. The actual surface height is then calculated by combining optical sectioning with phase unwrapping in the main cycle of the interference phase response, and the main cycle is determined using the bipolar property of differential confocal microscopy.

**Figure 4:**
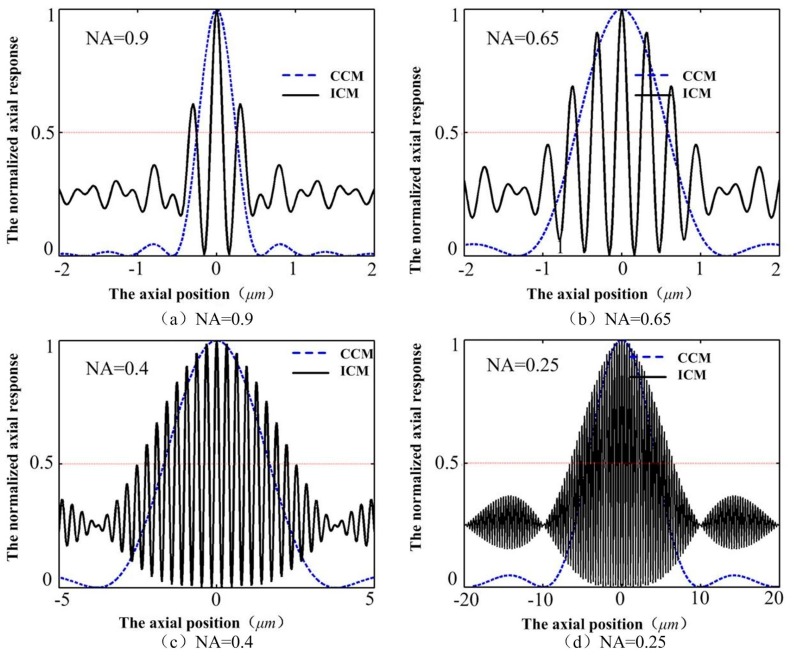
Comparison of normalized axial responses between conventional confocal microscopy (CCM) and Interference confocal microscopy (ICM) using single channel detection with NA = 0.9, 0.65, 0.4, and 0.25, respectively.

The axial depth resolution of conventional confocal microscopy (CCM) mainly depends on the NA of the objective lens, whose full width at half-maximum of the axial intensity response (FWHM) decreases with increasing numerical aperture of the objective lens. However, the FWHM of the ICM stays almost unchanged with the changing of the objective lenses (as shown in Figure 4), implying that we may obtain high resolution with low NA objective lenses, which can obtain large range and long working distance, in the ICM.

## 3. Results

**Figure 5:**
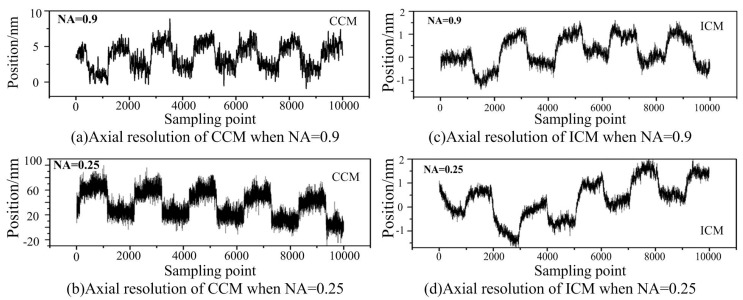
Comparison of the axial resolution between CCM and the ICM with different objective lenses.

To evaluate the axial resolution of the ICM, experiments are implemented with a 3D object translation stage, P-517.3CD (PI, with closed loop resolution 0.5 nm in the *z* axis and 1 nm in the *x*–*y* axis). The scanned object was a perfect reflector mirror mounted on the object stage. A series of reciprocating motions was generated axially by the stage in a typical workshop environment equipped with no excessive ground isolation for anti-vibration, which can be used to verify the validity of disturbance inhibition of the proposed ICM. Figure 5 shows the comparison of the axial resolution between the ICM and CCM with different objective lenses.

From Figure 5, we can see that the resolution observably decreases with the decreasing of the numerical aperture of the objective lens for conventional confocal microscopy, and suffers from environmental vibration. When NA = 0.9, the resolution of the conventional confocal microscopy is less than 1/10 of that when NA = 0.25. However, the resolution of the ICM stays almost unchanged with the changing of the objective lenses, and can be less affected by environmental vibration. The reason can be found according to Equation (2). Common-mode noises, such as the power fluctuation of the laser light and environmental vibration can be suppressed by the proposed method. The data drift was mainly due to the position drift of the object translation stage or the environmental vibration. Thus, the proposed ICM method is an effective way to image the object surface with reflectance disturbance and environment vibration.

It can also be clearly seen that the resolution of the proposed ICM is better than that of CCM, especially when the NA is low. Like other optical microscopes and profilers, the performance and capability of the proposed ICM is largely dependent on the lens objectives it uses. Objectives determine the magnification, working distance, slope capability, and field of view of the microscope (but not Z-height resolution, which is constant). The axial depth resolution of the ICM could reach 1 nm with the NA of the objective lens being 0.9 and 0.25. The working distance of the objective lens with NA = 0.25 is 10 mm, implying that we can obtain the combined axial depth resolution with a sub-nanometer to greater than 10 mm working distance even with a low-NA objective lens in ICM.

Furthermore, the Areal Star Pattern (ASG-0.2, serial number: NPL-BNT 010) developed by the National Physical Laboratory (NPL) are used for verification of the axial depth resolution (ISO/FDIS 25178 part 603 (2012)). The nominal height of the step on the ASG-0.2 is 200 nm. Three measurements were made, and the mean height of the three measurements calculated by NPL (Reference: 2014060310/1). The measured mean height of step is 185.1 nm with an expanded uncertainty of 2.3 nm at a coverage factor *k* = 2.0.

**Figure 6:**
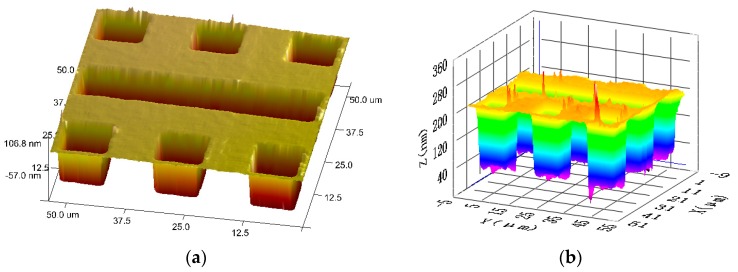
3D map of Areal Star Pattern. (**a**) 3D map tested by atomic force microscopy (AFM); (**b**) 3D map tested by ICM (DLL Plan Objective numerical aperture (NA) = 0.80).

**Figure 7:**
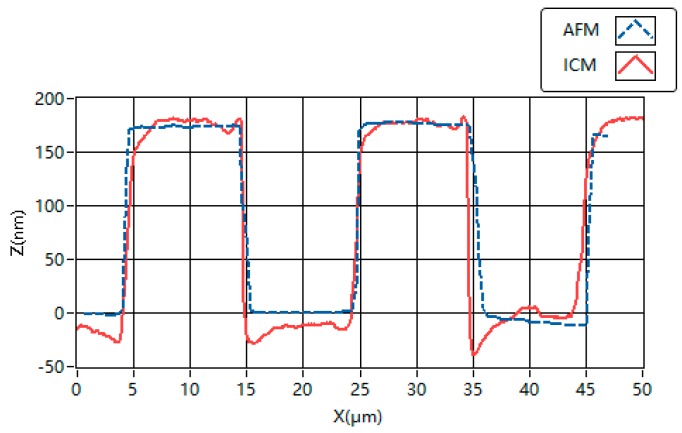
Profiles of the grooves obtained with AFM and the proposed ICM.

The comparison experiments of height measurements are implemented between atomic force microscopy (AFM) and the proposed ICM, as shown in Figure 6. The profiles of the grooves obtained with AFM (Bruker Dimension ICON and Nanoscope V8.10) and the proposed ICM are given in Figure 7 as a comparison. The glitches of the 3D map in Figure 7 may be caused by accidental error and system noise.

The profile of the steps is also measured and calculated in the NPL method. The profiles obtained by these two methods have similar distributions of height and width. The mean heights measured by AFM and the proposed ICM are 176.5 nm and 193.5 nm, respectively. Obviously, the test result of the proposed ICM is closer to the nominal height and the calibrated height by NPL than that of AFM.

## 4. Conclusions

An interference confocal microscope integrated with a new single-body four-step simultaneous phase-shifter device has been presented to obtain a large height dynamic range, high axis resolution, and reduce the sensitivity to vibration and reflectance disturbance seamlessly. The compact single body spatial phase-shifter is introduced to replace the previous complex phase-shifter devices and get a stable and space-saving, simplified system. The comparison experiments of resolution testing and height measurements have been implemented for verification of the axial depth resolution.
